# Laryngotracheobronchial papillomatosis: an uncommon cause of recurrent respiratory infection

**DOI:** 10.1590/0037-8682-0441-2019

**Published:** 2020-01-27

**Authors:** Bruno Niemeyer de Freitas Ribeiro, Edson Marchiori

**Affiliations:** 1 Hospital Casa de Portugal - Rede Casa/3D Diagnóstico por Imagem, Departamento de Radiologia, Rio de Janeiro, RJ, Brasil.; 2 Universidade Federal do Rio de Janeiro, Departamento de Radiologia, Rio de Janeiro, RJ, Brasil.

**FIGURE 1:**
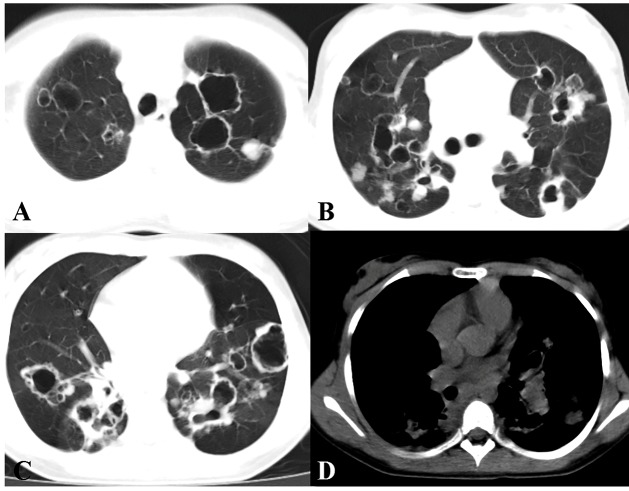
Chest computed tomography showing multiple cavitated nodules scattered throughout both lungs **(A-C)**, with no evidence of calcification **(D)**.

An 11-year-old female patient presented with chronic cough and recurrent respiratory infection, without other associated comorbidities. Laboratory test results were unremarkable. Chest computed tomography showed multiple cavitated nodules in both lungs lungs (Figure 1). Bronchoscopy revealed small nodular lesions in the trachea; histopathological analysis confirmed diagnosis of laryngotracheobronchial papillomatosis (LP).

LP, caused by human papillomavirus, is characterized by the appearance of papillomas in the aerodigestive tract, with pulmonary involvement in ~ 1% of cases^1^ ^,^ ^2^ ^,^ ^3^. Infection commonly occurs at birth, by passing through the infected mother’s birth canal^1^ ^,^ ^2^ ^,^ ^3^. The main clinical manifestations are hoarseness, cough, stridor, dyspnea, and recurrent infection. The course of the disease is unpredictable, ranging from spontaneous remission to pulmonary dissemination requiring multiple surgical interventions, and malignant transformation to squamous cell carcinoma of the lung^1^ ^,^ ^2^ ^,^ ^3^. Although this diagnosis may be suggested by clinical and radiological findings, final diagnosis is made by histopathological analysis. 
